# *Anopheles moucheti* and *Anopheles vinckei* Are Candidate Vectors of Ape *Plasmodium* Parasites, Including *Plasmodium praefalciparum* in Gabon

**DOI:** 10.1371/journal.pone.0057294

**Published:** 2013-02-20

**Authors:** Christophe Paupy, Boris Makanga, Benjamin Ollomo, Nil Rahola, Patrick Durand, Julie Magnus, Eric Willaume, François Renaud, Didier Fontenille, Franck Prugnolle

**Affiliations:** 1 Laboratoire MIVEGEC, UMR 224-5290 CNRS-IRD-UM1-UM2, IRD Montpellier, France; 2 Centre International de Recherches Médicales de Franceville (CIRMF), Franceville, Gabon; 3 Institut de Recherche en Ecologie Tropicale, Libreville, Gabon; 4 Parc de la Lékédi, ERMAET/COMILOG, Bakoumba, Gabon; Walter & Eliza Hall Institute, Australia

## Abstract

During the last four years, knowledge about the diversity of *Plasmodium* species in African great apes has considerably increased. Several new species were described in chimpanzees and gorillas, and some species that were previously considered as strictly of human interest were found to be infecting African apes. The description in gorillas of *P. praefalciparum*, the closest relative of *P. falciparum* which is the main malignant agent of human malaria, definitively changed the way we understand the evolution and origin of *P. falciparum*. This parasite is now considered to have appeared recently, following a cross-species transfer from gorillas to humans. However, the *Plasmodium* vector mosquito species that have served as bridge between these two host species remain unknown. In order to identify the vectors that ensure ape *Plasmodium* transmission and evaluate the risk of transfer of these parasites to humans, we carried out a field study in Gabon to capture *Anopheles* in areas where wild and semi-wild ape populations live. We collected 1070 *Anopheles* females belonging to 15 species, among which *An. carnevalei*, *An. moucheti* and *An. marshallii* were the most common species. Using mtDNA-based PCR tools, we discovered that *An. moucheti*, a major human malaria vector in Central Africa, could also ensure the natural transmission of *P. praefalciparum* among great apes. We also showed that, together with *An. vinckei*, *An. moucheti* was infected with *P. vivax*-like parasites. *An. moucheti* constitutes, therefore, a major candidate for the transfer of *Plasmodium* parasites from apes to humans.

## Introduction

Recent studies demonstrated that African great apes are infected by different *Plasmodium* species [1], [2], among which four species that are traditionally regarded as human parasites (*P. falciparum* (*3, 4*), *P. vivax* [3], [5], [6], *P. malariae* [5], [6] and *P. ovale* [7]. These findings and in particular the discovery in gorillas of parasites that are genetically very close to *P. falciparum* (these parasites were named *P. praefalciparum* [2]), have changed paradigms concerning the origin of *Plasmodium* in humans and emphasized the risk of cross-species exchanges between apes and humans [1], [2].

*Plasmodium* parasites are obligatory mosquito-borne pathogens and *Anophelinae* mosquitoes might have played and still play a crucial role in their transfer to humans [8]. Inter-human transmission of *Plasmodium* species is well characterized in sub-Saharan Africa [9]. Conversely, nothing is known about the vector species that ensure *Plasmodium* transmission in African apes. To identify such vectors, authors [10] analyzed mosquito specimens collected close to nests of wild chimpanzees in Western Uganda, but failed to detect any infected mosquito. Yet, several species, including the *Anopheles* (*An.*) *moucheti* “sub-species” and species from the *Anopheles nili* group, might be candidate vectors [2]. Indeed, these species are major human malaria vectors, are present in and around forests in West and Central Africa and their distribution range largely overlaps with that of great apes. To identify the vectors of ape *Plasmodium* and evaluate the risk of transfers to humans, we started a field study in Gabon to capture *Anopheles* specimens in close proximity to wild and semi-wild ape populations. DNA amplification was used to detect *Plasmodium*-positive specimens.

## Results and Discussion

**Figure 1:**
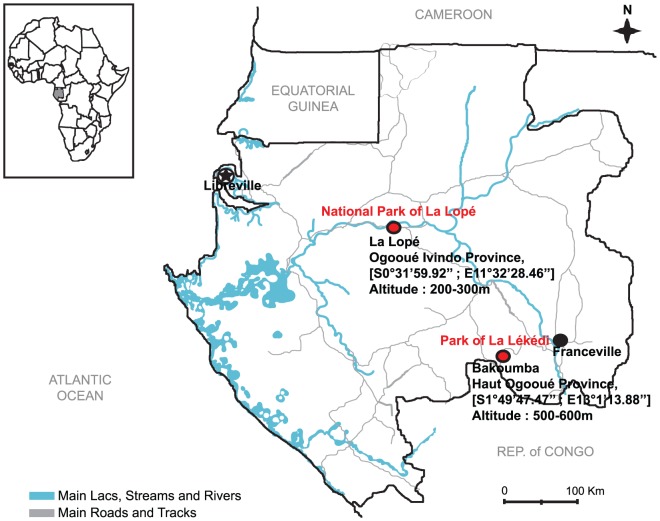
Location of the mosquito collection sites in Gabon.

Mosquito specimens were collected in the Park of La Lékédi (Bakoumba, Haut Ogooué Province) and the National Park of La Lopé (Ogooué Ivindo Province), in Gabon (Figure 1). Wild chimpanzees (*Pan troglodytes troglodytes*) and gorillas (*Gorilla gorilla gorilla*) live in these two wildlife natural reserves and they can be infected by ape *Plasmodium* (*P. gaboni*, *P. reichenowi* in Bakoumba [11] and *P. reichenowi*, *P. GorB*, *P. praefalciparum* in La Lopé (*Ollomo and Prugnolle, unpublished data*)). The Park of La Lékédi also hosts semi-wild chimpanzee and gorilla orphans in natural forest enclosures far from human dwellings. *Anopheles* specimens were captured using CDC light traps between October 2010 and April 2012 and identified by reference to standard morphological identification keys and by using molecular tools. Overall, 1070 female *Anopheles* specimens that belonged to at least 15 species were collected (Table 1). In the Park of La Lékédi, *An. moucheti moucheti* was the most prevalent species followed by *An. marshallii*, whereas in La Lopé, most of the specimens belonged to *An. carnevalei* (*nili* group).

**Table 1:** *Anopheles* mosquitoes collected in La Lékédi and La Lopé Parks between October 2010 and April 2012.

	Sites								
	La Lékédi					La Lopé			Both sites
*Collection dates* *Season*	08–13/11/2010Rainy (short)	08–13/04/2011Rainy (long)	07–09/12/2011Rainy (short)	24–27/04/2012Rainy (long)	All collection dates	24–27/10/2010Rainy (short)	25–30/03/2012Rainy (long)	All collection dates	
***Anopheles species***									
*An. coustani*	–	–	–	1	1	4	1	5	6
*An. eouzani*					–	1	–	1	1
*An. funestus*	3	–	–	–	3	15	–	15	18
*An. gambiae ss M form*	–	–	–	–	–	12	2	14	14
*An. jebudensis*	–	–	1	–	1	–	–	–	1
*An. maculipalpis*	–	–	–	–	–	–	1	1	1
*An. marshallii*	103	15	26	50	194	43	7	50	244
*An. moucheti moucheti*	197*	3	69**	1	270	3	1	4	274
*An. nili group*									
*An. carnevalei*	–	–	–	–	–	198	186	384	384
*An. nili ss*	3	0	2	1	6	–	–	–	6
*An. paludis*	10	3	13	1	27	–	–	–	27
*An. rodhesiensis rodhesiensis*	–	–	–	–	–	1	–	1	1
*An. theileri*	–	–	–	–	–	6	–	6	6
*An. tenebrosus*	–	–	3	–	3	–	–	–	3
*An. vinckei*	8	–	–	23**	31	–	–	–	31
*An. sp*	20	–	2	–	22	30	1	31	53
***All species***	**344**	**21**	**116**	**77**	**558**	**313**	**199**	**512**	**1070**

The table lists the number of females collected for each species. *An. sp*: when visual identification was not possible due to the deterioration of the morphological features.

* including one specimen infected with *P. praefalciparum.*

** including one specimen infected with *P. vivax-like.*

**Figure 2:**
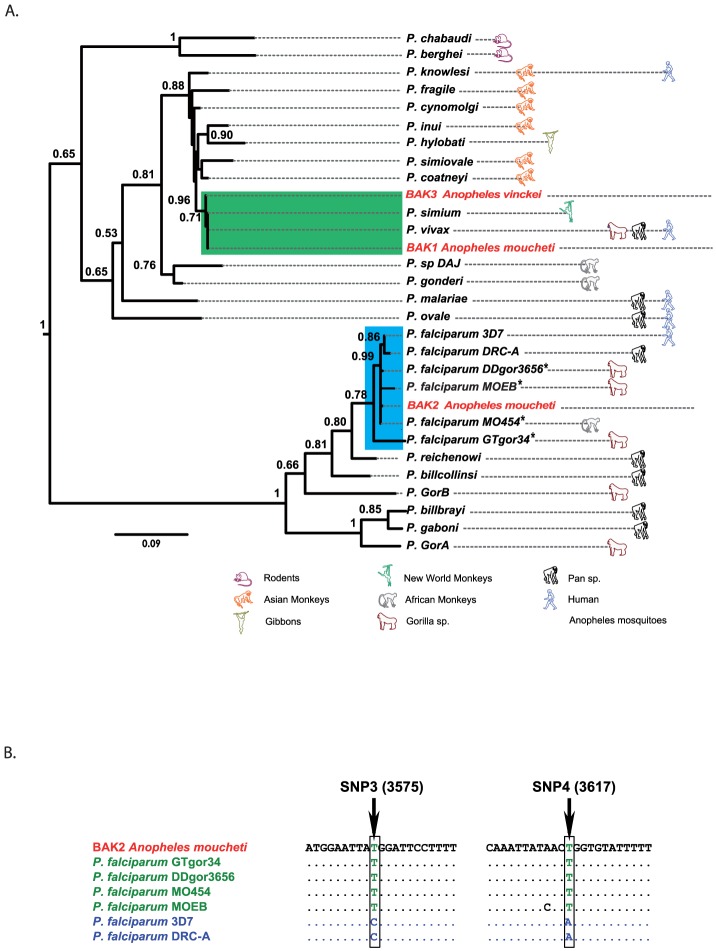
Phylogenic position of parasites infecting mosquitoes. A: Maximum-likelihood sub-tree of *Plasmodium* species obtained from the alignment analysis of 917 bp-long *cyt b* sequences. Bootstrap values are indicated at each node when >0.5. Avian *Plasmodium* sequences (*P. gallinaceum* and *P. juxtanucleare*) were used to root the tree. See the Material and Methods section and the supporting information file Table S1 for details and GenBank accession numbers of the different sequences. *Plasmodium falciparum* isolates marked with an asterisk were also designated as *P. praefalciparum* (2) B: Comparison of the diagnostic mitochondrial SNPs identified in the parasite DNA from the *BAK 2 An. moucheti* isolate with those present in *P. falciparum* infecting apes (*i.e., P. praefalciparum*) (in green) and humans (in blue) as described in [3], [14].

Total DNA was extracted from all mosquito specimens and used as template to screen for the presence of *Plasmodium* DNA by PCR amplification of a portion of the parasite cytochrome b (*cyt b*) gene. This assay showed that eleven specimens from La Lékédi Park and four from La Lopé were infected by *Plasmodium* parasites. Sequence analyses revealed that three DNA samples from specimens collected in Bakoumba (*BAK1 Anopheles moucheti*, *BAK2 Anopheles moucheti* and *BAK3 Anopheles vinckei*) contained DNA that belonged to primate *Plasmodium* species (*P. vivax*–like in “*BAK1 An. moucheti”* and “*BAK3 An. vinckei”* and a *P. falciparum*-like parasite in “*BAK2 An. moucheti”)* (Figure 2 A), whereas the parasite DNA detected in all the other positive samples was genetically related to *Polychromophilus sp*. (bat *Haemosporidia*). Analysis of two diagnostic SNPs (at position 3575 and 3617 of the mitochondrial genome) that allow differentiating between human *P. falciparum* and gorilla *P. praefalciparum* [3] revealed that the parasite identified in the “*BAK2 An. moucheti”* isolate was related to *P. praefalciparum* (Figure 2 B). As the mosquito collection sites in Bakoumba were all located far from human dwellings (>10 km), it is very likely that apes or other non-human primates were the source of these parasites.

The BAK1 *An. moucheti* specimen infected by a *P. vivax*-like parasite was collected close to a semi-wild group of seven chimpanzees in the natural forest enclosure of La Miula (GPS position: S1°46'54.66''; E12°58'42.53''). It is likely that these chimpanzees constituted the source of parasite infection, although we cannot exclude infection after mosquito feeding on wild animals that are frequently observed near this site. The *BAK3 An. vinckei* specimen, also infected by a *P. vivax*-like parasite, was collected close to recent wild gorilla nests (GPS position: S1°44'33.24''; E12°55'55.54''). The detection of this parasite confirms that sylvatic transmission of *P.vivax*-like parasites occurs in Africa [3], [5], [6] and raises the possible role of apes as reservoir of *P. vivax* in West and Central Africa, as previously suggested [5], [6]. Indeed, although in these regions 95%–99% of the population is resistant to *P. vivax* blood-stage infection because of the protective effect of Duffy-negative erythrocytes [12], *P. vivax* infections are frequently reported in travelers returning from these areas [13]. The existence of a sylvatic reservoir could explain these infections. Further analysis to compare apes and human *P. vivax* strains should be carried out to assess this hypothesis.

The *BAK2 An. moucheti* specimen (infected by *P. praefalciparum)* was trapped close to the sanctuary of three young gorillas (GPS position: S1°47'20.29''; E12°59'35.20'') that represent the most probable source of infection, although an infection from wild non-human primates cannot be excluded. *Plasmodium praefalciparum*, the closest known relative of *P. falciparum* in non-human primates, was first discovered in wild gorillas (hence the proposition that *P. falciparum* in humans is of gorilla origin [3]) and then also in a *Cercopithecus nictitans* in Gabon [14], thus suggesting that monkeys could also be a source of infection.

Our results suggest that *An. vinckei* and *An. moucheti* can support the sylvan transmission of *P. vivax* and *P. praefalciparum* in apes. Across all collection dates, the percentages of ape *Plasmodium* infected mosquitoes in Bakoumba were 0.74 and 3.22% respectively for *An. moucheti* and *An. vinckei.* As an indication, the sporozoïte rates among potential vectors estimated in two Gabonese villages where the malaria transmission is endemic were 0.6 and 2.7% [15]. Nevertheless, considering the methodology we used (parasite detection done on whole mosquito with an unsuited molecular tool to discriminate parasite stages), these results are not sufficient to demonstrate the effective vector transmission. To definitively confirm this hypothesis it will be necessary to detect the presence of sporozoites (vertebrate infecting stage) in mosquito salivary glands by using microscopic, immunologic or molecular tools, and also to design additional studies to determine the trophic behavior of these mosquito species. *Anopheles moucheti,* like mosquitoes of the *nili* group (*An. nili ss and An. carnevalei*), is present in the equatorial forests of Central Africa along river networks where their immature stages are usually found at the edge of rivers and streams [16]. *An. moucheti* is considered to be mainly anthropophilic in anthropogenized forest environments (i.e., villages) where it is a major human malaria vector [17]. Very little is known about its propensity to bite animals (and particularly non-human primates) because previous studies focused mainly on humans in rural and urban environments and never in sylvan environments where wildlife represents the main blood-source. *Anopheles moucheti* might actually be a zoo-anthropophilic vector that can readily operate *Plasmodium* transfers between apes and humans. Conversely, the role of sylvan *Anopheles* species, such as the strictly zoophilic *An. vinckei* [18], is probably restricted to the transmission of *Plasmodium* among apes.

## Conclusion

Here we identified two candidate vectors of *P. vivax*-like parasite and *P. praefalciparum* among apes in Africa. Our results suggest that *An. moucheti*, which is as a major *Plasmodium* vector in humans in Central Africa [17], might transmit *Plasmodium* in apes in the same geographic area and possibly also between apes and humans. Other *Anopheles* species, such as those from the *nili* group and *An. marshallii* that were previously implicated in human malaria transmission, were not infected by *Plasmodium*, but their density suggests a possible involvement. Further studies to gather entomological/parasitological and trophic behavior data are needed to solve the enigma of *Plasmodium* transmission in African apes and to evaluate the risk of transmission to humans at a time when contacts between humans and non-human primates are steadily increasing.

## Materials and Methods

### Collection of mosquito specimens

All mosquito collections were authorized by the Ministère de la Recherche Scientifique et du Développement Technologique du GABON (authorizations N° AR0009/10/MENERSI/CENAREST/CG/CST/CSAR and N° AR0006/12/MENERSI/CENAREST/CG/CST/CSAR). The "Agence Nationale des Parcs Naturels" (ANPN) and the "Centre National de la Recherche Scientifique et Technologique" (CENAREST) of Gabon authorized this study and facilitated the access to the National Park of la Lopé.

We consecutively collected mosquito specimens in two Gabon regions where great apes live and are known to be naturally infected by ape *Plasmodium* (*P. gaboni*, *P. reichenowi* in Bakoumba [11] and *P. reichenowi*, *P. GorB*, *P. praefalciparum* in La Lopé (*Ollomo and Prugnolle, unpublished data*)). The landscape in Bakoumba and la Lopé corresponds to a forest-savannah mosaic. The climate of Gabon is equatorial with two rainy seasons that extend from February to April and from October to December. In Bakoumba (Haut Ogooué Province), mosquitoes were sampled in the private Park of La Lékédi that houses wild and semi-wild (sanctuary for orphans) populations of chimpanzees (*Pan troglodytes troglodytes*) and gorillas (*Gorilla gorilla gorilla*) during four periods between November 2010 and April 2012. We also collected mosquito specimens in the National Park of La Lopé (Ogooué Ivindo Province) during two periods between October 2010 and March 2012. In this wildlife reserve, populations of both ape species are also present. *Anopheles* mosquitoes were captured using CDC light traps placed at ground level (1.5 m) in forest areas close to the ape sanctuaries (in the Park of La Lékédi) and in several places where great apes were susceptible to rest (nests and feeding areas) (Table 2). All sites (including the sanctuaries) were located far from human dwellings (up to 10 km in the Park of La Lékédi and up to 2 km in the La Lopé Park) with scarce human activities between 8:00 AM and 5:00 PM.

**Table 2:** Information on sampling organization at each site.

	Date of collection	Type of sites	Number of CDC traps
La Lopé National Park	24–27/10/2010, 25–30/03/2012	9 different sites where wild great apes live	32
Park of La Lékédi	08–13/11/2010, 08–13/04/2011, 07–09/12/2011, 24–27/04/2012	5 different sites where wild great apes live	32
		Chimpanzee sanctuary 1,La Miula	39
		Chimpanzee sanctuary 2, Lékédi Lake	8
		Gorilla sanctuary	28

Collected mosquitoes were morphologically identified by reference to standard morphological features [19], stored in liquid nitrogen, sent to the CIRMF and kept at −80°C until processed for molecular analyses.

### DNA extraction, PCR amplification and sequencing

DNA was isolated and purified from the whole mosquito body using the DNeasy Blood and Tissue kit (Qiagen) according to the manufacturer's instructions.

DNA from mosquito specimens belonging to the *An. gambiae*, *An. moucheti* and *An. nili* complexes was used for species identification by using PCR-based diagnostic tools according to previously described procedures [20], [21], [22].

To search for the presence of *Plasmodium* DNA, total extracted DNA was used as a template for amplifying a 950 bp fragment of the parasite cytochrome b (*cyt b*) gene with a nested PCR protocol that was previously described in [11]. At the end of the second round of amplification, PCR-amplified products (5 µL) were run on 1.5% agarose gels in TBE buffer, and positive products were sequenced by Eurofins (Germany).

### Phylogenetic analyses

We performed phylogenetic analyses using the obtained *cyt b* sequences and 27 previously published *cyt b* sequences from different *Plasmodium* species (see the supporting information file Table S1 for details concerning hosts and accession numbers). Multiple alignments of all partial *cyt b* sequences were carried out by using ClustalW (v 1.8.1 in BioEdit version 7.0.9.0) [23]. The maximum-likelihood (ML) tree construction was based on *cyt b* sequences of 917 nucleotides in length. The best-fitting ML model was identified according to the Akaike information criterion and by using ModelTest [24] and was a general time reversible model with gamma-distributed rates of variations (GTR +I+ Γ) for the nucleotides. The highest-likelihood DNA trees and corresponding bootstrap support values were obtained with PhyML (freely available through the ATGC bioinformatics facility http://www.atgc-montpellier.fr, [25]), by using nearest neighbor interchange (NNI) plus subtree pruning regrafting (SPR) branch swapping and 500 bootstrap replicates [26].

## Supporting Information

Table S1 **Parasite Cyt b sequences used in** **Figure 2** **.**
(DOC)
