# Supplementary material for: Anopheles moucheti and Anopheles vinckei Are Candidate Vectors of Ape Plasmodium Parasites, Including Plasmodium praefalciparum in Gabon
Source: PLoS One. 2013 Feb 20;8(2):e57294. doi: 10.1371/journal.pone.0057294 (PMC3577705; doi:10.1371/journal.pone.0057294)
Supplement: Table S1 — Parasite Cyt b sequences used in Figure 2 . (DOC) [file pone.0057294.s001.doc]

**Table S1.** Parasite Cyt b sequences used in Figure 2.

| **Name used in this study** | **Species** | **Recognized Host** | **Accession Numbers** |
| --- | --- | --- | --- |
| *P. gallinaceum* | *Plasmodium gallinaceum* | Birds | NC008288 |
| *P. juxtanucleare* | *Plasmodium juxtanucleare* | Birds | NC008279 |
| *P. chabaudi* | *Plasmodium chabaudi* | Rodents | AF014116 |
| *P. berghei* | *Plasmodium berghei* | Rodents | AF014115 |
| *P. knowlesi* | *Plasmodium knowlesi* | Asian Monkeys, Humans | AY722797 |
| *P. fragile* | *Plasmodium fragile* | Asian Monkeys | AY722799 |
| *P. cynomolgi* | *Plasmodium cynomolgi* | Asian Monkeys | AY800108 |
| *P. inui* | *Plasmodium inui* | Asian Monkeys | AB354572 |
| *P. hylobati* | *Plasmodium hylobati* | Gibbons | AB354573 |
| *P. simiovale* | *Plasmodium simiovale* | Asian Monkeys | AB434920 |
| *P. coatneyi* | *Plasmodium coatneyi* | Asian Monkeys | AB354575 |
| *P. vivax* | *Plasmodium vivax* | Gorillas, Chimpanzees, Humans | NC007243 |
| *P. simium* | *Plasmodium simium* | New World Monkeys | NC007233 |
| *P. sp DAJ* | *Plasmodium sp* | African Monkeys | AY800112 |
| *P. gonderi* | *Plasmodium gonderi* | African Monkeys | AB434918 |
| *P. malariae* | *Plasmodium malariae* | Chimpanzees, Humans | AB354570 |
| *P. ovale* | *Plasmodium ovale* | Chimpanzees, Humans | AB354571 |
| *P. GorA* | *Plasmodium sp*. gorilla clade G2 / KGgor1227 | Gorillas | HM235284 |
| *P. GorB* | *Plasmodium sp*. gorilla clade G3 / GTgor119 | Gorillas | HM235376 |
| *P. gaboni* | *Plasmodium gaboni* | Chimpanzees | FJ895307 |
| *P. billbrayi* | *Plasmodium billbrayi* | Chimpanzees | GQ355468 |
| *P. billcollinsi* | *Plasmodium billcollinsi* | Chimpanzees | GQ355479 |
| *P. reichenowi* | *Plasmodium reichenowi* | Chimpanzees | NC002235 |
| *P. falciparum GTgor34* | *Plasmodium falciparum* | Gorillas | HM235308 |
| *P. falciparum MO454* | *Plasmodium falciparum* | African Monkeys | JF923762 |
| *P. falciparum MOEB* | *Plasmodium falciparum* | Gorillas | JF923761 |
| *P. falciparum DDgor3656* | *Plasmodium falciparum* | Gorillas | HM235367 |
| *P. falciparum DRC-A* | *Plasmodium falciparum* | Chimpanzees | GQ355474 |
| *P. falciparum 3D7* | *Plasmodium falciparum* | Humans | AY282930 |
| *BAK1 Anopheles moucheti* | *Plasmodium vivax* | *Anopheles moucheti* | KC140104 |
| *BAK2 Anopheles moucheti* | *Plasmodium falciparum* | *Anopheles moucheti* | KC140103 |
| *BAK3 Anopheles vinckei* | *Plasmodium vivax* | *Anopheles vinckei* | KC140105 |
